# Small-Nucleic-Acid-Based Therapeutic Strategy Targeting the Transcription Factors Regulating the Vascular Inflammation, Remodeling and Fibrosis in Atherosclerosis

**DOI:** 10.3390/ijms160511804

**Published:** 2015-05-22

**Authors:** Sung Won Youn, Kwan-Kyu Park

**Affiliations:** 1 Department of Radiology, Catholic University of Daegu Medical Center, School of Medicine, Catholic University of Daegu, Daegu 705-718, Korea; E-Mail: ysw.badest.2010@gmail.com; 2 Department of Pathology, Catholic University of Daegu Medical Center, School of Medicine, Catholic University of Daegu, Daegu 705-718, Korea

**Keywords:** atherosclerosis, inflammation, remodeling, fibrosis, transcription factor, gene therapy, oligodeoxynucleotides

## Abstract

Atherosclerosis arises when injury to the arterial wall induces an inflammatory cascade that is sustained by a complex network of cytokines, together with accumulation of lipids and fibrous material. Inflammatory cascades involve leukocyte adherence and chemotaxis, which are coordinated by the local secretion of adhesion molecules, chemotactic factors, and cytokines. Transcription factors are critical to the integration of the various steps of the cascade response to mediators of vascular injury, and are induced in a stimulus-dependent and cell-type-specific manner. Several small-nucleic-acid-based therapeutic strategies have recently been developed to target transcription factors: antisense oligodeoxynucleotides, RNA interference, microRNA, and decoy oligodeoxynucleotides. The aim of this review was to provide an overview of these particular targeted therapeutic strategies, toward regulation of the vascular inflammation, remodeling and fibrosis associated with atherosclerosis.

## 1. Introduction

Despite lifestyle modifications and the use of new pharmacological agents to lower plasma cholesterol concentrations, atherosclerosis continues to represent a major health issue, since it is a significant cause of morbidity and mortality, and can lead to ischemia and infarction of the brain, heart, visceral organs, or extremities [1]. Atherosclerosis of the large and medium-sized elastic and muscular arteries affects about 12%–14% of the general population, and its prevalence increases significantly with age and risk factors including smoking, hypertension, diabetes, hyperlipidemia, homocysteinemia, and metabolic syndrome [2,3].

Atherosclerosis is a multifactorial and progressive disease with spectral pathophysiological features: disordered lipid metabolism, inflammation, and thrombosis [4]. Lipids and fibrous materials accumulate at the intimal and medial layers, but this constitutes more than the simple accumulation of lipids within the artery wall itself [5]. Chronic inflammation and arterial remodeling due to proliferative processes play important roles at all stages of the disease. Chronic vascular inflammation is a complex set of interactions among cytokines and cells that can arise in response to injury or endothelial dysfunction [6]. Activated endothelium expresses chemokines, including monocyte chemotactic protein (MCP)-1 and interleukin (IL)-8, and adhesion molecules, including intercellular adhesion molecule (ICAM)-1, vascular cellular adhesion molecule (VCAM)-1, and E- and P-selectin, leading to monocyte/lymphocyte recruitment and infiltration into the subendothelium. Along with early endothelial dysfunction and altered vascular smooth muscle cell (VSMC) contractility, the forming atheroma is a site of excessive production of cytokines and inflammatory ligands by various cell types that mediate inflammation and immune responses.

A key step in the development of atherosclerosis is the migration of circulating monocytes into the subendothelial space and their differentiation into macrophages. The circulating leukocytes are recruited into the vascular tissues through cellular adhesion and chemotaxis, and subsequent leukocyte activation, which are initiated by nonspecific vascular injury. Monocytes and neutrophils are among the early inflammatory cells to attach to the endothelium and move into the subendothelial space. Monocytes transform into macrophages and are further activated by encounters with various cytokines including tumor necrosis factor α (TNF-α), IL-1, and IL-6. As the lipid-rich plaque progresses, accumulating macrophages and other migrating cells, as well as activated endothelial cells (ECs), secrete proinflammatory cytokines, matrix metalloproteinases (MMPs), and cathepsins, causing plaque fragility. The cells within the plaque secrete matrixproteases that degrade the extracellular matrix (ECM) and make the fibrous cap fragile, leading to rupture and thrombus formation. The vascular remodeling and fibrosis occur as a result of lack of elastin and excessive deposition of ECM components such as fibronectin, type I collagen, and plasminogen activator inhibitor-1 (PAI-1) [7,8]. This proliferation of VSMCs and the accumulation of ECM lead to vascular fibrosis and increased arterial stiffening, reduced lumen diameter, and arterial wall thickening, with these intrusions into the lumen altering the flow of blood. Intimal hyperplasia is the thickening of the tunica intima of a blood vessel as a complication of a reconstruction procedure or endarterectomy. Intimal hyperplasia is the universal response of a vessel to injury and is an important reason of late bypass graft failure, particularly in vein and synthetic vascular grafts.

These intercellular networking and intracellular signal transductions are modulated by short nucleic acids, both transcriptionally and posttranscriptionally, among VSMCs, macrophages, T lymphocytes, and ECs. Hence, targeting short nucleic acids on transcription factors may be a feasible approach with which to inhibit target gene expression related to vascular inflammation, remodeling and fibrosis in the atherosclerosis, and intimal hyperplasia after the reconstruction procedure.

**Figure 1:**
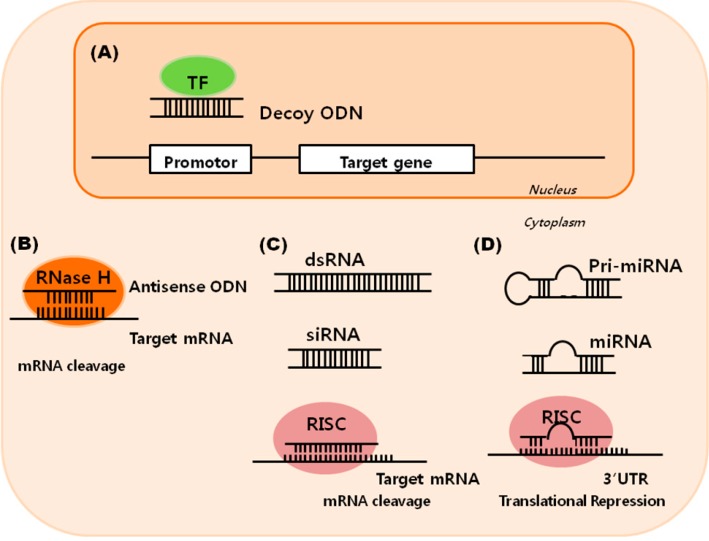
Small-nucleic-acid-based therapeutic strategies. (**A**) Double-stranded decoy oligodeoxynucleotides (ODNs) block the binding of transcription factors (TFs) with the promoter regions of target genes and inhibits specific gene expression at the pretranscriptional level; (**B**) Antisense ODNs specifically hybridize with their target messenger RNA (mRNA), which activates ribonuclease H (RNase H) that can degrade the target mRNA; (**C**) Small interfering RNA (siRNA) incorporate into an RNA-induced silencing complex (RISC) forming a perfect duplex with their target mRNA site, which leads to a specific cleavage of target mRNA; (**D**) microRNAs (miRNAs), which are small-non-protein-coding RNAs, are generated as primary RNA precursors of miRNAs (pri-miRNA) in the nucleus, and bind to the target 3ʹ untranslated (UTR) region. The regulation of gene expression at the posttranscriptional level occurs either by inhibiting the translation of protein from mRNA or by promoting the degradation of mRNA. dsRNA: double-stranded RNA.

The strategy of small nucleic acid therapy includes antisense oligodeoxynucleotides (ODNs), small interfering RNAs (siRNAs), microRNA (miRNA), and decoy ODNs (Figure 1). Decoy ODNs block the binding of the transcription factor and inhibit specific gene expression at the pretranscription level, while siRNAs, miRNAs, and antisense ODNs operate at the posttranslation level. Antisense ODNs specifically hybridize with their target messenger RNA (mRNA), activating ribonuclease H (RNase H) cleavage of the target mRNA. siRNAs form a perfect duplex with their target mRNA site, which leads to a specific cleavage of the target mRNA, and miRNAs are generated in the nucleus (primary RNA precursors of miRNAs; pri-miRNAs) and bind to the target 3ʹ untranslated region (UTR) through imperfect complementarity at multiple sites, which act on its target by translational repression or mRNA cleavage.

The aim of this review is to provide an overview of small-nucleic-acid-based therapeutic strategies targeted toward the transcription factors that regulate vascular inflammation, remodeling and fibrosis in atherosclerosis, and neointimal formation after surgical or interventional therapy (Table 1). The signal networks of the transcription factors as targets for controlling these vascular pathologies are also discussed.

**Table 1:** Small-nucleic-acid-based therapeutic strategies targeting the genes regulating vascular inflammation, remodeling and fibrosis in atherosclerosis, and neointimal formation after surgical or interventional therapy.

Strategy and Target Gene	Biological Effects	References
Antisense ODN		
ACE	The inhibition of ACE by antisense ODN attenuates neointimal formation	[9]
TGF	TβR-targeted antisense ODN inhibits the expression of TGF-β1, and local delivery of an antisense TGF-β1 construct inhibits intimal hyperplasia in autogenous vein grafts in rats	[10,11]
STAT	Antisense ODN targeting SOCS3 exacerbates the atherosclerotic process in apolipoprotein E(−/−) mice by increasing the size, leukocyte content, and chemokine expression in the lesions	[12]
siRNA		
TGF	TGF-β1 siRNA effectively reduces high-glucose-induced TGF-β1, PAI-1, and collagen type I mRNA, and protein expression in kidney and liver fibrosis	[13,14,15]
Ang II	Ang II induces ECM turnover and fibrosis via both TGF-β-dependent and -independent Smad3 signaling pathways. Ang II is involved in several intracellular signaling systems common to TGF-β, including activation of the Smad pathway, protein kinases (MAPK and ρ-kinase), and production of reactive oxygen species	[16,17,18,19]
Matrix metalloproteinase	RNA silencing targeted to matrix metalloproteinase-2 and -9 suppresses VSMC migration and neointimal formation in mice	[20,21]
VCAM-1	Transfection of mice aorta VSMCs with siRNAs targeting VCAM-1 results in a reduction in the number of migrated SMCs	[22]
JAK2/STAT3	IL-6, an inducer of the acute-phase response, induces the phosphorylation of STAT3, the proliferation of VSMCs, and the release of MCP-1. A specific JAK2 inhibitor AG490 partially inhibits STAT3 activation and MCP-1 production	[23]
STAT1	STAT1 is a point of convergence for proatherogenic IFN-γ and LPS-mediated TLR4-dependent signal transduction. A specific STAT1 inhibitor, fludarabine, inhibits IFN- and LPS-dependent adhesion of monocytes to ECs	[24,25,26]
c-Jun	The effect of IL-6 on P4Hα1 expression is mediated by c-Jun through the Raf-MEK1/2-ERK1/2 MAPK pathway, and silencing c-Jun with siRNA significantly reduces IL-6-induced plaque instability	[27]
NF-κB	siRNA knockdown of NF-κB inhibits the IL-17-mediated up-regulation of VCAM-1 expression	[28]
ERK1/2	ERK1/2 silencing attenuates IFN-induced ox-LDL uptake in macrophages and reverses high-glucose-induced CTGF-mediated proliferation and ECM production in VSMCs	[29,30]
CD74	CD74 siRNA decreases IFN-γ-induced NF-κB activation and MCP-1 production in VSMCs	[31]
TLR4	TLR4 silencing decreases ox-LDL-associated NF-κB activity, MCP-1, and IL-8	[32,33]
miRNA		
miR-21	miR-21 is increased in ligated or balloon-injured rat carotid arteries and in human atherosclerotic lesions	[34,35]
miR-143/145	miR-145 is the most abundant miRNA in VSMCs of normal rat carotid arteries	[36]
miR-146a	miR-146a targeting the KLF4 3ʹ UTR promotes VSMC proliferation *in vitro* and vascular neointimal hyperplasia *in vivo*	[37]
miR-126	miR-126 plays an antiatherogenic role by enhancing endothelial repair and repressing VCAM-1	[38,39]
miR-181	Overexpression of miR-181 inhibits importin-α3 expression and repression of NF-κB nuclear translocation, and subsequent repression of NF-κB-responsive genes such as those encoding the adhesion molecules VCAM-1 and E-selectin	[40,41]
miR-155	Expression of miR-155 is induced by TLR ligands such as LPS, mildly ox-LDL, and IFN-γ. miR-155 suppresses SHIP1, SOCS1, TGF-activated kinase 1/MAP3K7-binding protein 2, and Bcl-6. miR-155 also modulates TGF signaling in macrophages via the targeting of Smad2	[42,43]
Decoy ODN		
E2F	Double-stranded decoy ODNs corresponding to E2F binding sites block the activation of genes mediating cell-cycle progression, and inhibit VSMC proliferation and intimal hyperplasia in injured vessels	[44,45,46]
Sp1	Inhibition of the Sp1-binding promoter region was found to suppress Ang I receptor expression and decrease the blood pressure in rats	[47]
NF-κB	Treatment with a NF-κB decoy ODN reduces the activities of inflammatory cytokines such as TNF-α and IL-1β, and the expression of adhesion molecules was found to be reduced in an animal model	[48,49,50]
NF-κB + Sp1	Chimeric decoy ODN alleviates atherosclerotic changes and reduces serum cholesterol, inflammatory cytokines, and the expressions of atherosclerotic markers	[51]

ACE: angiotensin-converting enzyme; TGF: transforming growth factor; STAT: signal transducer and activator of transcription; SOCS: suppressors of cytokine signaling; siRNA: small interfering RNA; mRNA: messenger RNA; Ang: angiotensin; MAPK: mitogen-activated protein kinase; VCAM: vascular cellular adhesion molecule; VSMC: vascular smooth-muscle cell; MCP: monocyte chemotactic protein; JAK: janus kinase; Bcl-2: B-cell leukemia/lymphoma 2; CTGF: Connective-tissue growth factor; EC: Endothelial cell; ECM: Extracellular matrix; ERK: Extracellular-signal-regulated kinase; IL: Interleukin; IFN: Interferon; KLF: Krüppel-like factor; LPS: Lipopolysaccharide; MiRNA: MicroRNA; NF-κB: Nuclear factor κB; ODN: Oligodeoxynucleotide; ox-LDL: Oxidized low-density lipoprotein; P4Hα1: Prolyl-4-hydroxylase α1; PAI-1: Plasminogen activator inhibitor-1; SHIP1: Polyphosphate-5-phosphatase; SMC: Smooth-muscle cell; TLR: Toll-like receptor; TNF: Tumor necrosis factor; UTR: Untranslated region; TβR: TGF-β1 serine/threonine kinase receptor; SP1: Specificity protein 1.

## 2. Antisense Oligodeoxynucleotide (ODN) Strategy for Vascular Disease

Antisense ODNs are short chains of nucleic acids, usually comprising 10–30 nucleotides, which are typical single-stranded DNA or chemically modified DNA derivatives [52,53,54]. The base sequence is complementary to the target gene’s mRNA sense element, and antisense ODNs specifically hybridize with their complementary targeted mRNAs via Watson–Crick base-pairing. The sequence-specific binding of an antisense ODN to a target mRNA activates RNase H-cleavage of the target mRNA.

Various chemical modifications of antisense ODNs have been developed to prolong tissue half-life by enhancing nuclease resistance and to increase affinity and potency [54]. In the first generation with phosphorothioate (PS) modification, nonbridging oxygen atoms in the phosphodiester bond are replaced by sulfur atoms. In the second generation, 2ʹ-*O*-methyl or 2ʹ-*O*-methoxyethyl modifications of PS-modified antisense oligonucleotides (ASOs) are made. In the third generation, peptide nucleic acids, locked nucleic acids, and phosphoroamidate morpholino oligomers are the three most-studied ASOs.

### 2.1. Antisense ODN Targeted for Ang II

The antisense ODN is not only a useful experimental tool in protein-target identification and validation, but is also a potentially highly selective therapeutic strategy for atherosclerosis with dysregulated protein expression, as demonstrated in preclinical studies of angiotensin (Ang) II, transforming growth factor (TGF), and Janus kinase (JAK)/signal transducer and activator of transcription (STAT). Angiotensin-converting enzyme (ACE) and Ang II are involved in the remodeling of large and resistance arteries during hypertension, and inflammatory and tissue responses, and matrix remodeling are regulated by signaling events downstream of the Ang II type 1 receptor (AT1) [9,55]. Morishita *et al.* demonstrated that the inhibition of vascular ACE expression by antisense ODN results in the attenuation of neointimal formation [9].

### 2.2. Antisense ODN Targeted for TGF-β1

TGF-β is a pleiotropic cytokine with important effects on processes of vascular inflammation and fibrosis [4,6,7,8,56,57]. TGF-β1 signaling occurs via transmembrane serine/threonine kinase receptors, which are composed of receptor I (TβRI) and receptor II (TβRII). Exogenous antisense TβRI and TβRII can be used to block the TGF-β1 signaling pathway. TβR-targeted antisense ODN was shown to inhibit the expression of TGF-β1 [10], and local delivery of an antisense TGF-β1 construct inhibited intimal hyperplasia in autogenous vein grafts in rats [11].

### 2.3. Antisense ODN Targeted for JAK/STAT

It has recently been demonstrated that suppressors of cytokine signaling (SOCS) modulate JAK/STAT-mediated cellular responses during atherosclerosis [12,58,59]. It is reported that overexpression of SOCS suppressed STAT activation and reduced inflammatory gene expression and cell growth, whereas SOCS knockdown by antisense ODN increased these cellular responses. Antisense ODN targeting SOCS3 exacerbated the atherosclerotic process in apolipoprotein E(−/−) mice by increasing the size, leukocyte content, and chemokine expression in the lesions [12]. Conversely, treatment with SOCS1-derived peptide limited the development and progression of atherosclerosis in diabetic mice, and altered plaque composition and inflammation [59].

### 2.4. Clinical Trial of Antisense ODN Strategy

More than 40 clinical trials of antisense ODN agents are currently ongoing at various phases. The first antisense drug, fomivirsen, was approved by the US Food and Drug Administration for the treatment of cytomegalovirus-induced retinitis in AIDS patients [60]. With respect to vascular application, the recent clinical trial of mipomersen for hyperlipidemia is producing promising outcomes [61,62,63,64]. Mipomersen is a second-generation antisense ODN designed to inhibit apolipoprotein B-100 protein synthesis in the liver and the subsequent release of apolipoprotein B-containing lipoproteins into the circulation. The efficacy and safety of mipomersen added to maximally tolerated lipid-lowering therapies were demonstrated in the phase 3 study of patients with familial hypercholesterolemia and having or at a risk for coronary artery disease.

## 3. siRNA Strategy for Vascular Disease

siRNAs silence their target genes by binding to their complimentary mRNA and triggering their elimination [53,54,65,66]. However, this mechanism of RNA interference (RNAi) is slightly different to the antisense strategy. Long stretches of double-stranded RNA (dsRNA) can interact with the RNase-III-like enzyme Dicer to be cleaved into siRNA or noncoding dsRNA with a length of 21–23 nucleotides. One strand of the siRNA duplex, termed the guide strand, is incorporated into a nuclease-containing multiprotein complex called RNA-induced silencing complex (RISC), whereas the second, passenger strand, is released and degraded. Once incorporated into the RISC, the guide strand guides this complex to the target mRNA and induces endonucleolytic cleavage of complementary targets. This cleavage leads to rapid degradation of the entire mRNA molecule as a result of the generation of its unprotected RNA ends, whereas the RISC complex is recovered for further cleavage cycles.

In the real world, siRNA encounters challenge in order to optimize outcomes. siRNA molecules have a very short half-life *in vivo* and need to be chemically protected from thermal and enzymatic degradation. *In vivo* transfection efficiency is a vital determinant of target gene silencing, and poor uptake by cells may be another obstacle to overcome. Cell-specific siRNA delivery may decrease the amount of siRNA needed and mitigate unwanted off-target effects [67,68,69]. The off-target effects of siRNA are due to nonspecific siRNA/mRNA hybridization that adversely alters cell signaling. For example, p38 mitogen-activated protein kinases (MAPKs) are members of the MAPK family that respond to stress stimuli and play a role in cell differentiation and apoptosis. However, the application of long dsRNA in vertebrates is limited because it induces a generalized suppression of protein synthesis and cell death by activating the interferon (IFN) pathway. The proper half-life, transfection efficiency, and cell-specific delivery need to be considered further in order to facilitate clinical translation.

### 3.1. siRNA Targeted for TGF-β/Smad Signaling Pathways

TGF-β1 plays an important role in vascular remodeling and fibrosis [4,8,56,57,70], and TGF-β activation via Smad signaling up-regulates the transcription factor of several genes that are important for ECM formation, such as those encoding procollagens, fibronectin, connective-tissue growth factor (CTGF), and PAI-1 [16,71,72]. The inactive form of TGF-β is produced bound to latency-associated peptide, which is anchored in the ECM by binding with latent TGF-β-binding protein (LTBP). The proteolytic cleavage of latent TGF-β–LTBP–ECM into the biologically active TGF-β is activated by thrombospondin-1, plasmin, acidic microenvironments, MMPs (MMP-2 and MMP-9), and β6 integrin [56,57,73].

TGF-β1 transmits its signals predominantly via cytoplasmic proteins called Smads, which act as transcription factors that translocate into the nucleus [56,57,70]. Upon TGF-β1 engagement, TβRII with serine/threonine kinase activity phosphorylates TβRI, which in turn phosphorylates Smad2/3 on two serine residues at their *C*-terminus, enabling binding to Smad4 to form heteromeric Smad complexes. TGF-β1 increases the phosphorylation of Smad2 and Smad3, which form heterotrimers with Smad4. The receptor-regulated Smad (R-Smad)-common mediator Smad (Co-Smad) complexes translocate into the nucleus and bind to Smad-related DNA sequences, where transcription factors participate in the regulation of target gene expression for vascular fibrosis, such as fibronectin, type I collagen, and CTGF.

The TGF-β signaling pathway is inhibited by binding to the type I receptors (Smad7) or by competing with activated R-Smad1 for binding to Co-Smad4 (Smad6). Smad7 inhibits the synthesis of TGF-β-induced fibronectin, type I collagen, and CTGF [7,74]. Vascular Smad7 overexpression attenuates remodeling and neointima formation after balloon injury in rat carotid arteries [74], and may reduce the incidence of restenosis [75].

Based on these obseravations, TGF-β1 RNAi may be a feasible therapeutic option for vascular remodeling and fibrosis. TGF-β1 siRNA effectively reduces high-glucose-induced TGF-β1, PAI-1, and collagen type I mRNA, and protein expression in kidney and liver fibrosis [13,14,15], and its application in vascular pathologies deserves further investigation.

### 3.2. siRNA Targeted for Ang-II-Induced Signaling Pathways

Ang II induces ECM turnover and fibrosis via both TGF-β-dependent and -independent Smad3 signaling pathways [16,17,18,19]. Ang II is involved in several intracellular signaling systems common to TGF-β, including activation of the Smad pathway, protein kinases (MAPK and ρ-kinase), and production of reactive oxygen species [76,77,78,79,80,81,82,83]. Ang-II-induced tubular CTGF and type I collagen expressions were up-regulated by phosphorylated Smad2 or Smad3 but down-regulated by deletion of TGF-β1, overexpression of Smad7, or knockdown of Smad2 or Smad3 [16,18]. Ang II increases the protein levels of Smad2 and Smad4 in association with increased TGF-β and PAI-1 levels in diseased vessels in VSMCs. Over-expression of Smad7, which interferes with the receptor-mediated activation of Smad2, reduces Ang-II-induced CTGF up-regulation, and fibronectin and type-1 procollagen over-expression. The AT1 antagonist losartan reduces Ang-II-induced Smad activation, which has been demonstrated to exert beneficial effects on vascular fibrosis and remodeling by interfering with both TGF-β synthesis and Smad activation [18]. Ang II also stimulates TGF-β secretion, which in turn triggers ECM synthesis. Ang-II-induced ECM synthesis was found to be inhibited by a TGF-β neutralizing antibody or truncated TGF-β type II receptor [19].

Ang-II-induced signaling pathways are also mediated by p38 MAPK and ρ-associated protein kinase activation [73,76,77,78,79,80,81,82,83]. The p38 MAPK inhibitor SB203580 was shown to reduce Ang-II-induced Smad2 phosphorylation, and p38 MAPK silencing was found to reduce the accumulation of low-density lipoprotein (LDL)-induced cholesterol in macrophages and prevent LDL-cholesterol-loading-induced inhibition of autophagy [82]. p38 MAPK silencing inhibited both Ang-II- and TGF-β1-induced peroxisome proliferator-activated receptor γ (PPARγ) reduction [83]. PPARγ ligands have been shown to ameliorate Ang-II-induced atherosclerotic changes, and Ang II suppresses PPARγ expression and activity in VSMCs in a TGF-β1-dependent fashion.

### 3.3. siRNA Targeted to Matrix Metalloproteinases (MMPs)

Activation of MMPs and their isoforms leads to ECM remodeling, which facilitates VSMC-driven neointimal formation [84,85,86,87]. RNA silencing targeted to MMP-2 and MMP-9 was found to suppress VSMC migration and neointimal formation in mice [20,21]. Furthermore, MMP-9 silencing in ECs can provide cell-protective effects by ameliorating high-glucose-induced damage [88].

### 3.4. siRNA Targeted to Adhesion Molecules

Interfering VCAM-1 expression could be an effective approach toward the prevention and treatment of atherosclerosis and restenosis [22,89,90]. VCAM-1 is an adhesion molecule that is expressed by ECs for recruitment of leukocytes during inflammation. It is also abundantly expressed by VSMCs in atherosclerotic lesions and in injured arteries. In one study, VSMCs were isolated from the aorta of C57BL/6 mice and transfected with siRNAs targeting VCAM-1; this resulted in a significant reduction in the number of migrated smooth-muscle cells (SMCs) [22].

### 3.5. siRNA Targeted for JAK/STAT

JAK/STAT system consists of three main components: a receptor, JAK, and STAT [91,92,93]. Binding of the ligand to the receptor triggers the activation of JAKs. JAKs with tyrosine kinase activity bind to cytokine receptors, and then STAT protein binds to the phosphorylated receptor, where the cytosolic inactive STAT is phosphorylated by JAKs. Active STAT dimers translocate into the nucleus and bind to a promoter, which controls the transcription of their target genes [93].

The role of the JAK/STAT pathway in the development of vascular remodeling was recognized from IL-6-induced MCP-1 production in rat VSMCs [23]. IL-6, an inducer of the acute-phase response, induced the phosphorylation of STAT3, the proliferation of VSMCs, and the release of MCP-1. A specific JAK2 inhibitor AG490 partially inhibited STAT3 activation and MCP-1 production, demonstrating that the JAK2/STAT3 pathway partially mediates IL-6-induced MCP-1 production. Zhang *et al.* showed that IL-6 can destabilize atherosclerotic plaques by down-regulating collagen content via a reduction in prolyl-4-hydroxylase α1 (P4Hα1) expression, which is required for collagen synthesis [27]. The effect of IL-6 on P4Hα1 expression is mediated by c-Jun through the Raf-mitogen-activated protein kinase kinase (MEK)1/2-extracellular-signal-regulated kinase (ERK)1/2 MAPK pathway, and silencing c-Jun with siRNA significantly reduces IL-6-induced plaque instability.

IFN-γ plays a role in atherosclerosis via JAK/STAT1 signaling [24,25,26]. IFN-γ sensitizes ECs to lipopolysaccharide (LPS)-induced STAT1 phosphorylation and the expression of target genes. In VSMCs, STAT1 is a point of convergence for proatherogenic IFN-γ and LPS-mediated toll-like receptor (TLR)4-dependent signal transduction. IFN, TLR, and IL-6 were shown to augment VSMC and leukocyte migration, leukocyte-to-EC adhesion, and foam-cell formation. Furthermore, IFN-γ and TLR4-activated signaling inhibit IL-6 STAT3-dependent anti-inflammatory signaling and potentially shift IL-6 to a STAT1-activating pro-inflammatory cytokine [25,26]. Therefore, STAT1 could be a promising therapeutic target in atherosclerosis. A specific STAT1 inhibitor, fludarabine, inhibits IFN and LPS-dependent adhesion of monocytes to ECs. However, the inhibition of STAT1 in vascular remodeling is hampered by the strong toxicity of purine nucleosides.

In THP-1 macrophage-derived foam cells, IFN-γ down-regulates ATP-binding cassette transporter (ABCA)1 expression and cholesterol efflux through the JAK/STAT pathway [94]. The JAK/STAT pathway is activated by IFN-γ, and STAT1 dimers bind to the gamma activated sequence element in the nucleus, down-regulating liver X receptor (LXRα). STAT1 siRNA compensates for the down-regulation of IFN-γ on ABCA1 expression and cholesterol efflux. LXRα-specific activation by an LXRα agonist almost compensated for the down-regulation of ABCA1 expression by IFN-γ, while siRNA of LXRα produced a greater down-regulation [94].

### 3.6. siRNA Targeted to Nuclear Factor-κB and Its Associated Noncanonical Transcription Factors

Nuclear factor (NF)-κB is a modulator of inflammation that plays a critical role in injury response and vascular diseases [95,96,97,98,99,100,101], and NF-κB itself or its associated transcription factors can be an RNAi target. Zhang *et al.* showed that IL-17-induced VCAM-1 expression in VSMCs is dependent on NF-κB [28]. siRNA knockdown of NF-κB was shown to inhibit the IL-17-mediated up-regulation of VCAM-1 expression. At the same time, IL-17-induced expression of VCAM-1 is partially dependent on the activation of ERK1/2 [102]. ERK is also integral to the IFN-γ-mediated activation of STAT1 and the expression of key genes implicated in atherosclerosis [29,30,102]. ERK1/2 silencing was found to attenuate IFN-induced oxidized LDL (ox-LDL) uptake in macrophages [29] and reverse high-glucose-induced CTGF-mediated proliferation and ECM production in VSMCs [30]. IFN-γ-induced NF-κB activation and MCP-1 production in VSMC was decreased by CD74 siRNA [31]. CD74 levels are increased in atherosclerotic plaques and peripheral blood monocytes in patients with carotid stenosis and are associated with intima-media thickness in subjects free from clinical cardiovascular diseases. TLR4 may provide a pathophysiological link between lipids and infection/inflammation and atherosclerosis. TLR4 silencing decreased ox-LDL-associated NF-κB activity, MCP-1, and IL-8 [32], and attenuated the ox-LDL-stimulated nuclear translocation of NF-κB, and MAPK phosphorylation [33].

### 3.7. Clinical Trial of siRNA Strategy

Most current literatures on the siRNA technology present results from the *in vitro* experiment and animal model, and the clinical trial using siRNA strategy on the vascular disease is still not on the road. To overcome limitations of half-life, transfection efficiency, and off-target effect, local application in diaseased arterial segments might be one of feasible option. In conjunction with surgical or interventional treatment, siRNA may be considered to be coated on the surface of vascular stents and bypass graft materials. Another feasible option may be encapsulation of siRNA into delivery vehicle such as lipid nanoparticle formulation. Recent clinical trials of encapsulated RNAi in lipid nanoparticle for transthyretin amyloidosis, caused by the deposition of hepatocyte-derived transthyretin amyloid in peripheral nerves and the heart, have demonstrated safety and efficacy with respect to lowering transthyretin levels [103].

## 4. miRNA Strategy for Vascular Disease

Recent work by several investigators has revealed that miRNA can control vascular pathologies and this is now recognized as another type of transcription factor [104,105]. miRNA represents a class of highly conserved, single-stranded, noncoding, small RNAs that control cellular function and are usually 20–25 nucleotides long. The regulation of gene expression at the posttranscriptional level occurs either by inhibiting the translation of protein from mRNA or by promoting the degradation of mRNA [104]. However, the control of miRNA-dependent gene expression is rather complex—More than one miRNA can cooperatively bind to the same 3ʹ UTR [104,106]. One miRNA is able to regulate the expression of multiple genes due to its ability to bind to its mRNA targets as either an imperfect or perfect complement. As a group, miRNAs may directly regulate at least 30% of the genes in a cell, and are therefore involved in the regulation of all major functions.

Regulation of miRNA biogenesis represents an important site of regulation of miRNA activity, since it involves multiple overlapping interactions between proteins and RNA [107]. miRNA genes have promoters that bind transcription factors that regulate their expression, similar to protein-coding genes, and various cofactors of Dicer, Drosha, and related compounds regulate the production of miRNAs, augmenting the miRNA processing machinery. miRNAs are produced from longer primary RNA precursors (pri-miRNAs) that contain stem-loop structures that are transcribed by RNA polymerase II. The Drosha-DGCR8 complex cleaves pri-miRNA into hairpin-loop structural precursor miRNAs (pre-miRNAs) of approximately 70 nucleotides in length. Pre-miRNAs are transported into the cytoplasm by exportin-5 and processed by the nuclease Dicer into the 20–24-nucleotide mature miRNAs. The guide strand (mature miRNA) is incorporated into a RISC and binds to the 3ʹ UTR of the target mRNA. This leads to either translational repression or degradation of the target mRNA.

The miRNA expression profile differs significantly between atherosclerotic plaques and control arteries, and the most up-regulated miRNAs are involved in processes known to be connected to atherosclerosis; for example, the miRNAs miR-21, miR-210, miR-34a, and miR-146a/b were shown to be up-regulated in human atherosclerotic plaques in the Tampere Vascular Study [108]. More recent studies found that several miRNAs, such as miR-10a, miR-126, miR-145, miR-146a/b, miR-185, miR-210, and miR-326, were expressed in plaques associated with atherosclerosis [109]. It has been demonstrated that some of these miRNAs (e.g., miR-143/145 and miR-126) are protective during vascular remodeling, whereas others (e.g., miR-21) may promote the cellular response that leads to neointima formation [108,109].

### 4.1. miR-21

The expression of miR-21 is increased in ligated or balloon-injured rat carotid arteries and in human atherosclerotic lesions [34,35]. TGF-β and bone morphogenetic protein signaling promote a rapid increase in the expression of mature miR-21 through a posttranscriptional step, thereby promoting the processing of primary transcripts of miR-21 into precursor miR-21 by the Drosha complex [110]. Expression of miR-21 in SMCs induced a synthetic phenotype by repression of specificity protein 1 (Sp1)/cystathionine lyase (CSE)-mediated SMC differentiation [111]. The expression of miR-21 repressed Sp1 protein expression by directly targeting the Sp1 3ʹ UTR regions, which in turn down-regulated CSE mRNA expression and stimulated VSMC proliferation. It has been found that phosphatase and tensin homolog (PTEN) and B-cell leukemia/lymphoma (Bcl)-2 are also involved in miR-21-mediated cellular effects; increased expression of miR-21 following vascular injury promotes neointimal growth, probably by suppressing PTEN, a potent negative regulator of the phosphoinositide-3-kinase/protein kinase B signaling pathway [111] and up-regulating Bcl-2 [34].

In ECs, miR-21 induced by high shear stress was found to suppress PTEN [112], while low shear stress has also been demonstrated to up-regulate miR-21; these mechanisms constitute a positive feedback loop that regulates oscillatory shear stress (OSS)-induced endothelial inflammation [113]. The induction of miR-21 by OSS represses PPAR through direct targeting at its 3ʹ UTRs. Decreased expression of PPAR reduces the inhibitory effects of PPAR on the activation of activator protein-1 (AP-1), and hence promotes the expression of adhesion molecules VCAM-1 and MCP-1 and EC inflammation, as well as miR-21 transcription. The increase in miR transcripts would further repress PPAR, thereby constituting a positive feedback circuit. In a recent study, it was suggested that TGF-β-induced endothelial-to-mesenchymal transition (EndMT) is at least partially mediated by miR-21, given that miR-21 blockade by transfection of specific microRNA inhibitors was found to partly prevent TGF-β-induced EndMT [114].

### 4.2. miR-143/145 and miR-146a

In response to shear stress, Krüppel-like factor (KLF)2 increases miR-143/145 in ECs, and triggers their microvesicle-mediated transfer to VSMCs, which confers atheroprotection. miR-145 is the most abundant miRNA in normal rat carotid arteries that is selectively expressed in VSMCs and phenotypic regulators of VSMCs [36]. miR-143/145 can direct the fate and function of smooth muscle to regulate the quiescent *vs.* proliferative VSMC phenotype [115]. Vascular injury decreases miR-145 expression in VSMCs, which in turn increases KLF5 expression and vascular neointimal formation [36]. The effects of miR-145 and miR-143 converge on serum response factor-dependent transcription by regulation of coactivators and corepressors to dictate the proliferative or differentiated VMSC phenotype.

Sun *et al.* demonstrated that miR-146a targets the KLF4 3ʹ UTR and plays an important role in promoting VSMC proliferation *in vitro* and vascular neointimal hyperplasia *in vivo* [37]. Transfection of an antisense miR-146a ODN into balloon-injured rat carotid arteries markedly decreased the progression of neointimal hyperplasia. The silencing of miR-146a in VSMCs increases KLF4 expression, whereas overexpression of miR-146a decreases KLF4 levels, showing that KLF4 competes with KLF5 to bind to and regulate the miR-146a promoter, and KLF4 and KLF5 exert opposing effects on the miR-146a promoter. Overexpression of KLF4 in VSMCs decreases miR-146a transcription levels, forming a feedback loop to regulate each other’s expression and VSMC proliferation.

### 4.3. miR-10

miR-10 contributes to regulation of the endothelial phenotype observed in atherosclerosis formation through the NF-κB signaling pathway [116,117]. miR-10 expression is lower at atherosusceptible arterial sites than at atheroprotected sites in swine aorta [117]. Knockdown of miR-10 in ECs decreases the expression of mitogen-activated kinase kinase kinase 7 (MAP3K7 or TAK1) and β-transducin repeat-containing gene, which up-regulates the phosphorylation of nuclear factor of κ light polypeptide gene enhancer in B-cell inhibitor (IκB)α, causing greater nuclear transport of NF-κB p65 and up-regulation of inflammatory cytokines such as MCP-1, IL-6, IL-8, VCAM-1, and E-selectin [117,118].

### 4.4. miR-24

Gene expression patterns are found to be different between early and advanced atherosclerotic plaque. miR-24, miR-155, miR-145, and miR-100 were active in early carotid lesions, which could be protective in the context of plaque evolution because they were not active in advanced plaques [119].

In macrophages, miR-24 controls an invasive subset of macrophages and retards in atherosclerotic plaque progression by modulating MMP-14 expression. In human coronary atherosclerotic plaques, increased MMP-14 protein expression in foam cell macrophages was associated with unstable lesions [120]. miR-24 expression in atherosclerotic plaques was inversely related to MMP-14 protein expression. Stable plaques contained higher miR-24 levels than unstable plaques, and miR-24 colocalized with foam cell macrophages that exhibited low MMP-14 protein expression. Silencing miR-24 increased MMP-14 expression and enhanced invasive capacity of macrophage, promoting proteolytic activity and instability of atherosclerotic plaque.

In the murine models and the patients of abdominal aortic aneurysm (AAA), miR-24 is a key regulator of vascular inflammation and AAA development [121]. AAA progression was associated with down-regulation of the miR-23b-24-27b cluster in murine AAA models, and Human AA also display miR-24 down-regulation, correlating inversely with aneurysm size. Chitinase 3-like 1 (Chi3l1) is a major target and effector under the control of miR-24 involved in vascular inflammation. Chi3l1 regulates cytokine synthesis in macrophages, promoting aortic smooth muscle cell migration and cytokine production, and stimulating adhesion molecule expression in vascular endothelial cells.

On the other hand, miR-24 is not entirely protective as seen from the phenotypic switch of VSMC from quiescent “contractile” to proliferative “synthetic”. miR-24 is induced by PDGF-BB, which in turn leads to down-regulation of Tribbles-like protein-3 (Trb3). Repression of Trb3 coincides with reduced expression of Smad proteins and decrease in BMP and TGFβ signalling, promoting a synthetic phenotype in VSMCs [122].

### 4.5. miR-181b

Proinflammatory TNF-α, IL-1β, and LPS increase a set of adhesion molecules in ECs, which recruit inflammatory cells to the site of inflammation [123]. This induction of adhesion molecules is mediated mainly by the NF-κB pathway [95,96,97,98,99,124]. Among the many different homodimers and heterodimers in the NF-κB/Rel family, the p50/p65 heterodimer is predominant in ECs [97]. In resting ECs, NF-κB binds to IκB protein, an inhibitor protein of NF-κB, and is localized in the cytoplasm [125]. Once ECs are activated, the IκB kinase complex is phosphorylated, rapidly degrading IκBα by the 26S proteasome. This leads to the immediate translocation of NF-κB heterodimers into the nucleus, following induction of several of the inflammatory response genes. In response to these proinflammatory stimuli, miR-181 regulates NF-κB-mediated EC activation and vascular inflammation by targeting importin-α3, a protein that is required for the nuclear translocation of NF-κB [40,41]. Over-expression of miR-181 inhibits importin-α3 expression and repression of NF-κB nuclear translocation, and subsequent repression of NF-κB-responsive genes such as those encoding the adhesion molecules VCAM-1 and E-selectin. Systematic administration of miR-181 mimics reduced leukocyte accumulation and EC activation in LPS-induced lung injury.

### 4.6. miR-126

miR-126 plays an antiatherogenic role by enhancing endothelial repair. The anti-inflammatory effects of miR-126 are demonstrated by the repression of VCAM-1, which reduces leukocyte adhesion to ECs *in vitro* [38,39]. This anti-inflammatory effect of miR-126 appears to be restricted to a subset of ECs, as demonstrated by the finding that miR-126-mediated repression of VCAM-1 upon inflammatory stimulation occurs in the glomerular and sinusoidal ECs of the liver, but not in the heart or lung [126]. The transcription of miR-126 in EC is regulated by several transcriptional factors, such as E26 transformation-specific (Ets)-1, Ets-2, and KLF2 [39,127].

### 4.7. miR-221/222

Recent studies have shown that the biological functions of miR-221/222 in vascular walls are cell-specific [128,129]. miR-221/222 exhibits pro-proliferation, promigration, and antiapoptosis effects in VSMCs, but antiproliferation, antimigration, and proapoptosis effects in ECs. The differences in the expression profiles of their target genes—Those encoding p27(Kip1), p57(kip2), and c-kit—between the two cell types might be related to these opposing effects [128]. In advanced lesions, there was an increased proliferation rate of ECs lining vessels associated with a reduced expression of miR-222. Dentelli *et al.* identified STAT5A as a target of miR-222, and demonstrated that the expression of this microRNA was negatively correlated with STAT5A expression in human ECs from advanced neovascularized atherosclerotic lesions [129].

### 4.8. miR-155

miR-155 is one of the important miRNAs modulating the inflammatory response in macrophages and ECs [130]. In macrophages, the expression of miR-155 is induced by TLR ligands such as LPS, mildly ox-LDL, and IFN-γ, and IL-10 has the ability to promote anti-inflammatory gene expression by inhibiting miR155 [131]. This inhibitory effect of IL-10 on miR-155 was shown to lead to an increase in the expression of the miR-155 target, inositol polyphosphate-5-phosphatase (SHIP1). miR-155 suppresses SHIP1, SOCS1, TGF-activated kinase 1/MAP3K7-binding protein 2, and Bcl-6 [42]. miR-155 also modulates TGF signaling in macrophages via the targeting of Smad2 [43]. The sustained overexpression of miR-155 results in a decrease in Smad2, which in turn leads to a reduction in TGF-β-induced Smad2 phosphorylation and endogenous levels of Smad2 protein, thus altering the cellular responses to TGF-β by changing the expression of a set of genes involved in inflammation and fibrosis.

### 4.9. Clinical Trial of miRNA Strategy

As far as we can search, no clinical trial was found using miRNA strategy on the proliferative vascular disease. However, miRNA signatures are being applied currently in human clinical trials and miRNA-directed therapy is under way, with miR-122 targeting in hepatitis C being the most developed therapy thus far [132]. As seen from numerous preclinical investigations, miRNAs have potentials to be a new therapeutic target for proliferative vascular diseases. Besides miRNA has advantage to clarify both the molecular pathogenesis and the inherent complexities related to various vascular disease. Interfering with the miRNA expression in the artery wall is a potential method of affecting the development of atherosclerotic plaques and vascular disease, which requires future clinical translation. An increased understanding of fundamental miRNA biology, improved bioinformatics, and directed *in vivo* targeting to minimize off-target effects and toxicity will be required for successful translational application.

## 5. Decoy ODN Strategy for Vascular Disease

Gene expression is a complex biological process initiated by interactions between transcription factors and DNA. Transcription factors are proteins residing in the cell nucleus or cytoplasm that upon activation bind with specific DNA motifs in the promoter region of the target gene. Specific short DNA sequences bearing the consensus binding site bind the transcription factor and compete with promoter regions in a highly specific manner, and have been used successfully as “decoys” to bind specific transcription factors [133,134]. The occupation by a decoy ODN renders the associated transcription factors incapable of subsequent binding to the promoter region of target genes. It has been shown that this approach is effective in modulating gene expression *in vitro* and *in vivo*.

### 5.1. Decoy ODN Targeting E2F

Decoy ODNs blocking the activation of genes that mediate cell-cycle progression are effective in inhibiting VSMC proliferation [44,45,46,135]. The transcription factor E2F plays a pivotal role in the coordinated transactivation of genes regulating the cell cycle—such as *c-myc*, *c-myb*, *cdc2*, *PCNA* (encoding proliferating cell nuclear antigen), and *TK* (encoding thymidine kinase)—That are involved in lesion formation after vascular injury. A critical element in the regulation of cell-cycle progression involves the complex formed by the interaction between E2F, cyclin A, and cyclin-dependent kinase 2. E2F up-regulates the expression of these multiple genes, helping to speed up the parts of the cell cycle involving DNA synthesis and mitosis. The transfection of *cis*-element double-stranded decoy ODNs corresponding to E2F binding sites blocks the activation of genes mediating cell-cycle progression and inhibits the proliferation of VSMCs and neointimal hyperplasia in injured vessels.

### 5.2. Decoy ODN Targeting Sp1

The transcription factor Sp1 is a member of the Sp1/KLF-like family of zinc-finger transcription factors, and binds specifically to the guanine-cytosine (GC)-rich promoter region [136]. A GC-box-related sequence is located within the 58- to 34-base-pair region of the Ang I receptor gene promoter. The inhibition of Sp1 binding sites was found to suppress Ang I receptor expression, and thus decreased the blood pressure in rats [47,137]. Sp1 is also involved in the basal expression of ECM genes and may be important in the processes underlying fibrosis. In the fibrosis of organs such as liver, kidney, and lung, the expressions of ECM and inflammatory cytokines were decreased by using the Sp1 decoy ODN [138,139,140,141,142,143,144]. The Sp1 decoy ODN effectively blocks Sp1 binding to the promoter region for the transcription regulation of TGF-β1, and suppresses the expression of TGF-β1 and fibronectin. These studies suggest that an Sp1 decoy ODN could be used to alleviate vascular fibrosis.

### 5.3. Decoy ODN Targeting NF-κB

Activation of the transcription factor NF-κB was identified in VSMCs, macrophages, and ECs in atherosclerotic lesions [96,97,98,99]. NF-κB is activated in response to Ang II, ox-LDL, CD40 ligand, advanced glycation end products, and proinflammatory cytokines (TNF-α, IL-1, and IL-18), as well as following activation of the TLRs by LPS. In ECs, active NF-κB induces the transcription of cell adhesion molecules (e.g., ICAM-1, VCAM-1, and E-selectin), MMP-1, MMP-3, and MMP-9, tissue factors, and several cytokines (e.g., MCP-1, macrophage colony-stimulating factor, granulocyte-macrophage colony-stimulating factor, IL-1 IL-6, IL-8, and TNF-α) [6].

As an approach to inactivate NF-κB and reduce intimal hyperplasia, synthetic double-stranded DNA with a high affinity for NF-κB was introduced as a “decoy” cis element, to bind the transcription factor and block its expression [48,49,50,145]. *In vivo* transfection of this NF-κB decoy ODN inhibited neointimal formation and reduced the expressions of ICAM and VCAM in balloon-injured rat carotid arteries. Treatment with a ring-type NF-κB decoy ODN reduced the activities of inflammatory cytokines such as TNF-α and IL-1β, and the expression of adhesion molecules was reduced in the LPS/fat-induced mice [50], which exhibited similar features to those shown for the fibrotic response of organs such as liver or kidney [146,147].

### 5.4. Decoy ODN Targeting Two or More Transcription Factors

The chimeric decoy ODN, which contains two or more transcription-factor binding sequences in a single decoy molecule, was designed to enhance the effectiveness of the decoy ODN strategy. Several authors have demonstrated the feasibility of chimeric decoy ODN targeting simultaneously for NF-κB and E2F, NF-κB and E26 Ets, AP-, and Smad, NF-κB and Sp1, and Smad and Sp1 in inflammation and fibrotic lesions of various tissues [51,148,149,150,151,152,153]; it is expected that these results will extend to vascular fibrosis.

### 5.5. Ring-Type Decoy ODN

The ring-type (or closed-dumbbell) structure of decoy ODNs—Containing the consensus transcription factor binding sequence in a single decoy molecule without an open end—Has advantages over the conventional phosphorothioated ODN in that it is more stable and resistant to degradation by nucleases. Several authors have demonstrated the feasibility of the ring-type ODN decoy design for E2F, Sp1, NF-κB, and Ets [142,146,150].

### 5.6. Clinical Trial of Decoy ODN Strategy

Initial two clinical cases was presented on the use of a NF-κB decoy at the site of coronary stenting for the prevention of restenosis. There was no restenosis and systemic adverse effects during the periods of six months follow-up interval [154]. A subsequent open-label phase I/IIa clinical trial suggested the safety and effectiveness of NF-κB decoy ODN to prevent restenosis after percutaneous coronary intervention, demanding further placebo control trials [155].

In the PREVENT single-centre, randomised, controlled trial, human vascular bypass grafts were intraoperatively transfected *ex vivo* with E2F decoy ODN [156]. Mean transfection efficiency was 89.0%, and decreased significantly proliferating-cell nuclear antigen, c-myc mRNA concentrations and bromodeoxyuridine incorporation. At 12 months, fewer graft occlusions, revisions, or critical stenoses were seen in the E2F-decoy group than in the untreated group. However, the PREVENT III multicenter, randomized trial included 1401 patients showed no significant difference between the treatment groups in the end points, primary graft patency, or limb salvage [157]. Therefore *ex vivo* treatment of lower extremity vein grafts with E2F-decoy did not confer protection from reintervention for graft failure.

## 6. Conclusions

The inflammatory cascades of atherosclerosis are mediated by a myriad of transcription factors and their related signaling pathways. Together with the advanced understanding of its cellular, molecular, and genetic aspects, several strategies for small-nucleic-acid-based treatment have evolved targeting antisense ODNs, siRNAs, miRNA, and decoy ODNs. Despite several technical obstacles, the results of the small number of clinical trials conducted thus far are encouraging, and future clinical translation is to be expected.

## Abbreviations

ABCA: ATP-binding cassette transporter
ACE: Angiotensin-converting enzyme
Ang: Angiotensin
AP-1: Activator protein-1
AT1: Angiotensin II type 1 receptor
ASO: Antisense oligonucleotide
Bcl-2: B-cell leukemia/lymphoma 2
Co-Smad: Common mediator Smad
CSE: Cystathionine lyase
CTGF: Connective-tissue growth factor
dsRNA: Double-stranded RNA
EC: Endothelial cell
ECM: Extracellular matrix
EndMT: Endothelial-to-mesenchymal transition
ERK: Extracellular-signal-regulated kinase
Ets: E26 transformation-specific
GC: Guanine-cytosine
ICAM: Intercellular adhesion molecule
IFN: Interferon
IκB: Nuclear factor of κ light polypeptide gene enhancer in B-cell inhibitor
IL: Interleukin
JAK: Janus kinase
KLF: Krüppel-like factor
LDL: Low-density lipoprotein
LPS: Lipopolysaccharide
LTBP: Latent transforming-growth-factor-β-binding protein
LXRα: Liver X receptor α
MAPK: Mitogen-activated protein kinase
MCP: Monocyte chemotactic protein
MEK: Mitogen-activated protein kinase kinase
miRNA: MicroRNA
MMP: Matrix metalloproteinase
mRNA: Messenger RNA
NF-κB: Nuclear factor κB
ODN: Oligodeoxynucleotide
RISC: RNA-induced silencing complex
RNAi: RNA interference
ox-LDL: Oxidized low-density lipoprotein
P4Hα1: Prolyl-4-hydroxylase α1
PAI-1: Plasminogen activator inhibitor-1
PDGF: Platelet-derived growth factor
PPAR: Peroxisome proliferator-activated receptor
Pre-miRNA: Precursor microRNA
Pri-miRNA: Primary RNA precursor of microRNA
PS: Phosphorothioate
PTEN: Phosphatase and tensin homolog
RNase H: Ribonuclease H
R-Smad: Receptor-regulated Smad
SHIP1: Polyphosphate-5-phosphatase
siRNA: Small interfering RNA
SMC: Smooth-muscle cell
SOCS: Suppressors of cytokine signaling
Sp1: Specificity protein 1
STAT: Signal transducer and activator of transcription
TβRI: TGF-β1 serine/threonine kinase receptor I
TβRII: TGF-β1 serine/threonine kinase receptor II
TGF: Transforming growth factor
TLR: Toll-like receptor
TNF: Tumor necrosis factor
UTR: Untranslated region
VCAM: Vascular cellular adhesion molecule
VSMC: Vascular smooth-muscle cell
